# Endurance Training in Normobaric Hypoxia Imposes Less Physical Stress for Geriatric Rehabilitation

**DOI:** 10.3389/fphys.2017.00514

**Published:** 2017-07-20

**Authors:** Stephan Pramsohler, Martin Burtscher, Martin Faulhaber, Hannes Gatterer, Linda Rausch, Arn Eliasson, Nikolaus C. Netzer

**Affiliations:** ^1^Hermann Buhl Institute for Hypoxia and Sleep Medicine Research Bad Aibling, Germany; ^2^Department of Sport Science, University of Innsbruck Innsbruck, Austria; ^3^Department of Medicine, Uniformed Services University of the Health Sciences Bethesda, MD, United States; ^4^Division of Sports Medicine and Rehabilitation, Department of Medicine, University of Ulm Ulm, Germany

**Keywords:** hypoxia, training with geriatrics, low external load, training, geriatrics

## Abstract

**Rationale:** Evidence suggests that training in hypoxia can be very effective even while requiring less physical effort. We therefore aimed to measure the effect of endurance training under hypoxic conditions on pulmonary and cardiovascular parameters in an elderly population undergoing inpatient rehabilitation.

**Methods:** Forty patients over age 65 years with multiple co-morbid conditions were recruited during a 3-week stay in a geriatric rehabilitation center. Using a randomized, single-blinded, placebo-controlled design, patients were assigned to a hypoxic (HG) or normoxic (NG) group. HG patients completed seven training sessions of 30 min duration on a treadmill in a normobaric chamber with inspired oxygen fraction (FiO_2_) of 15.27%, with 10–30 min active training. Training was conducted with target heart rate at 80% of peak oxygen consumption (VO_2_-peak). NG group performed similar training in sham hypoxia (room air or FiO_2_ = 20.94%). At pre- and post-test completion, measures included: (1) cycle ergometry with ECG monitoring and measurement of VO_2_-peak, and (2) echocardiography for ejection fraction.

**Results:** The physical effort required of patients to reach target heart rate was reduced significantly (−28%, *p* = 0.043) in the HG compared to NG. Cardiopulmonary parameters showed no differences between groups.

**Conclusion:** Endurance training at 3,000 meters elevation imposes less stress on the locomotor systems while resulting in a similar physiological strain (i.e., heart rate). Hypoxic training holds promise for successful geriatric rehabilitation by being more accommodating to physical limitations in geriatric patients.

**Trial registration:** Registration at DRKS. (Approval No. 359/12, Trial No. DRKS00005241).

## Introduction

With the aging population, rehabilitative training for the elderly is gaining humanitarian and economic importance (Poterba, 2016). A major goal is to keep the elderly as fit and active as possible to prevent immobility and hospitalization (Jamour et al., 2014). This effort has the potential to improve quality of life and decrease burdens on the medical system. Despite intensive investigations into methods for training and therapy, there is a paucity of data and evidence-based guidelines for endurance training in the elderly ≥75 years (Meusel, 2000). However, endurance training does show positive results (Meusel, 2000; Oster et al., 2005; Fiogbe et al., 2017; Pandey et al., 2017). For common diseases such as coronary heart disease, stroke, cancer and diabetes, endurance training has proven to be effective in younger individuals <75 years (Buchner et al., 1997; Cornelissen and Fagard, 2005; Ventura-Clapier et al., 2007). Since those diseases have a high prevalence among geriatric patients, the question is raised regarding the benefit of such endurance training in the elderly. Restrictions in endurance capacity have a direct impact on immobility, muscle atrophy and frailty, all of which relate to activities of daily living (Avila-Funes et al., 2011). Recent studies in the field of endurance training suggest a greater effect of training at higher intensities (Hayashi et al., 2005; Swain and Franklin, 2006; Pokan et al., 2009). Higher intensities of training are especially hard to reach with geriatric patients because of physical and medical limitations. High-intensity training is even harder in rehabilitation settings such as after surgery. At times tolerance of active training may be reduced to 10–20 min per day. For endurance training to provoke adaptive responses in the cardiopulmonary systems in such a short time period, it may be necessary to conduct the training at 60–80% of peak oxygen (O_2_) consumption (VO_2_-peak) (Hayashi et al., 2005; Swain and Franklin, 2006; Pokan et al., 2009). Attaining this intensity level of training in the elderly may not be possible without substantial risk (Oster et al., 2005).

Positive effects of hypoxic training have been shown in prior investigations notably in obese subjects (Piehl Aulin et al., 1998; Netzer et al., 2008; Schipfer et al., 2008; Haufe, 2010; Gatterer et al., 2014). Initially, endurance performance in hypoxia is reduced, but even when applying a lower external load, such hypoxic training can result in cardiovascular and metabolic responses that are similar to high-intensity training in normoxia (Kong et al., 2017).

These observations suggest that hypoxic training may be useful to increase training intensity for geriatric patients while maintaining a low impact on locomotor systems. Hypoxic training may also have positive effects on ejection fraction (EF), heart rate (HR) to power ratio, O_2_ consumption (VO_2_), and revascularization of the myocardium (Knuth, 2008). In this study our aim was to measure the effect of endurance training under hypoxic conditions on pulmonary and cardiovascular parameters in an elderly population undergoing inpatient rehabilitation. We hypothesized that endurance training in normobaric hypoxia may induce a lower physical stress for a similar physiological strain compared to similar training in normoxia. As such, this may safely support targeted endurance training while respecting physical restriction in geriatric patients.

## Methods

Subjects were recruited from the geriatric rehabilitation hospital (Fachklinik Ghersburg für Geriatrische Rehabilitation, Bad Aibling, Germany) attached to the study center. Inclusion criteria for participation were: (1) age > 65 years, (2) cognitive ability to give informed consent and to participate in the study, (3) sufficient peripheral blood flow for pulse oximetry, (4) heart failure symptoms no worse than NYHA Class III, and (5) dosages of cardiovascular-relevant medications (ß-blockers, ACE inhibitors, etc.) that would allow at least a 10% increase of HR to physical stress. An exclusion criterion was any orthopedic condition that precluded performance of a cycle ergometry test.

Of the 40 patients recruited, 35 (87.5%) were able to complete the trial. Reasons for dropout were unwillingness to continue (*n* = 4) and termination for medical reasons (*n* = 1).

Descriptive means for the normoxic group (NG, *n* = 16) were: age 82.0 (±7.8) years, sex 11 women/5 men, and BMI 25.8 (±6.2) kg/m^2^. Descriptive means for the hypoxic group (HG, *n* = 19) were: age 80.2 (±7.2) years, sex 12 women/7 men, and BMI 25.2 (±5.3) kg/m^2^.

### Exercise testing

At pre-test, anthropometric data were collected and echocardiography was performed. Cycle ergometry with ECG monitoring was conducted using a standard protocol for heavily impaired patients (Pokan et al., 2009). After 3 min of rest and 3 min of reference pedaling at 15 watts, the test started at 27 watts and was increased by 7 watts each minute until subjective exhaustion (Pokan et al., 2009). Criteria that would trigger interruption of the test were: (1) HR higher than maximal predicted, calculated using the formula 220 bpm—age, (2) systolic blood pressure (BP) higher than 220 mmHg, (3) any ST-segment changes, (4) ventricular extrasystoles, or (5) angina pectoris. The testing was also interrupted upon attaining subjective exhaustion equivalent to 18 on the BORG-Scale (Borg, 1982). After pre-test, patients were randomly assigned in equal numbers to the HG or the NG.

### Training phase

The subsequent target HR for training was set as 80% of VO_2_-peak (maximum O_2_ consumption achieved during the pre-test ergometry test) with 5 bpm added to account for the conversion from cycle to treadmill training (Kroidl et al., 2015). The more standard conversion factor of 10 bpm was not used because patients most commonly train with their arms resting on the treadmill handrails. The main parameters that were used to assess training effects were EF by echocardiogram, peak HR, O_2_ saturation, VO_2_-peak, and peak O_2_/HR. Patients were asked to perform 7 training sessions during their 3-week stay in the geriatric rehabilitation center. The normobaric hypoxic chamber was set to contain either hypoxic air equivalent to an altitude of 3000 meters (F_i_O_2_ = 15.27%) or sham hypoxia (room air at F_i_O_2_ = 20.94%). Normobaric hypoxia was produced using a device called “Low Oxygen Systems” (Berlin-Buch, Germany) which provided a controlled mixture of fresh air and nitrogen to keep O_2_ and carbon dioxide (CO_2_) levels constant. This allowed patients to exercise without wearing a mask and to concentrate fully on the intervention. Patients were blinded to the different conditions though the investigators were not (single-blinded). Each of the 7 training sessions in the chamber included a stay of at least 30 min with a minimum of 10 min and a maximum of 30 min of active training on a treadmill. The treadmill (h/p/cosmos, Traunstein, Germany) offered forearm supports for more impaired patients and a safety belt to stop the movement in an emergency. The therapists tried to reach the target HR considering the physical and medical impairments of each patient and taking care not to exceed the target HR. BP (Omron M400 IT) was measured before each training session, while HR (Polar Heart Rate Sensor T31) and O_2_ saturation (NONIN Go 2) were monitored continuously during training.

The intervention protocol was designed to be practicable and achievable. The prescription of seven sessions of at least 10 min of endurance training over a 3-week period corresponded to common practice in geriatric rehabilitation hospitals. Both HG and NG subjects also participated in the other prescribed rehabilitation programs in the hospital, consisting of one 30-min individual physical therapy session and another 30-min group physical therapy session (e.g., strength, gait, balance training) per day. The mean time elapsed between training sessions as well as pre- and post-tests was 1.56 days in HG and 1.57 days in NG. At the conclusion of the 7 sessions of endurance training, outcome measurements were repeated as they had been performed at pre-test.

## Statistics

Evidence from published research suggested that for the main outcome parameter of VO_2_-peak, we would detect a mean difference of 0.23 l/min (SD ± 0.36 l/min) between intervention group and control group (Vaitkevicius et al., 2002; Vogt et al., 2002). To reach a significance level set a priori at *p* < 0.05 and a power of 80%, it would be necessary to enroll 17 subjects in each arm of the study. To account for possible dropouts, the study enrollment was set at 40 subjects in total. Data are presented as means ± standard deviation (SD). For all analyses a two-way ANOVA for repeated measures was performed. To identify the magnitude of statistical difference between the groups in each training session (Tr1-Tr7) a student *t*-Test was used. Data analyses were performed with the SPSS statistical software package (PASW Statistics for Windows version 21.0, SPSS Inc., Chicago, IL, USA). Effect size, using ANOVA, was calculated as partial eta-squared (η^2^). η^2^ < 0.06 accounts for a small effect, 0.06 < η^2^ > 0.14 accounts for a medium effect and η^2^ > 0.14 accounts for a big effect.

## Study approval

The study protocol was reviewed and approved by the institutional review board of the University of Ulm on the January 19, 2015 and was registered as a clinical trial with the German Clinical Trials Register (DRKS). (Approval no. 359/12, Trial no. DRKS00005241) Participants gave their written informed consent prior to inclusion in the study.

## Results

Thirty five patients (23 women) were able to finish the trial. Among these patients the most common diagnoses were coronary heart disease, diabetes types I and II, hypertension, chronic obstructive pulmonary disease, peripheral arterial occlusive disease, hip replacement, fractures, congestive heart failure, osteoporosis, stroke and myocardial infarction. The majority of patients were prescribed cardiovascular-relevant medications as well as diuretics, anticoagulant medication, cholesterol and blood sugar lowering drugs. During the study period, slight changes in medication were made by the hospital's physicians but these medication changes occurred in both groups equally.

The endurance training was well tolerated and there were no complaints regarding the intervention. For some patients, transfer to the cycle ergometer for ergospirometry was hard to accomplish. The gas analyzer mask was reported to be uncomfortable but tolerable for the short testing period.

### Outcome measurements before and after the intervention

Statistical analysis showed no significant differences between groups. From pre- to post-test, there were no changes in anthropometric parameters (Table 1). There was also no change in the measured cardiologic parameters. Mean EF for the HG showed a non-significant decrease compared to the NG. Although not significant, peak VO_2_ was higher in post-test in both groups. The peak O_2_ /-HR was higher in HG but this difference was not statistically significant. However, the maximum performance increased significantly in both groups from pre- to post-test (*p* = 0.004) (Table 1). There were no sex differences regarding the effect of the intervention. Overall, both groups showed similar responses from pre- to post-test though the training intensity (% of peak VO_2_) was significantly lower in HG (*p* = 0.012) (Table 2).

**Table 1:** Anthropometrical and physiological values at baseline (pre-) and after (post-) the endurance training for the hypoxic group (HG) and normoxic group (NG).

**Item**	**Hypoxic group (HG)**		**Normoxic group (NG)**		**Condition**	**Time**	**Interaction**
	**Pre-**	**Post-**	**Pre-**	**Post-**	***P***	***p* (η^2^)**	***p***
Weight (kg)	67.74 (±18.92)	68.21 (±19.34)	68.38 (±16.49)	67.78 (±17.75)	0.56	0.608	0.254
BMI (kg/m^2^)	25.23 (±5.26)	25.41 (±5.39)	25.83 (±5.71)	25.64 (±6.43)	0.457	0.827	0.287
EF (%)	42.83 (±9.28)	39.87 (±9.31)	46.61 (±15.70)	44.2 (±9.46)	0.493	0.169	0.931
Peak Power (W)	41.18 (±13.53)	46.19 (±14.43)	43.38 (±10.41)	46.24 (±11.41)	0.737	0.004^**^ (0.43)	0.289
Peak HR (bpm)	120.05 (±30.12)	120.37 (±24.48)	116.56 (±22.92)	120.31 (±20.41)	0.296	0.118	0.459
Peak VO_2_ (ml/min)	929.68 (±325.6)	1003.37 (±342.02)	938.13 (±218.01)	969 (±296.74)	0.858	0.375	0.703
Peak O_2_/HR (ml)	8.86 (±2.83)	9.4 (±3.14)	8.61 (±2.54)	8.66 (±2.59)	0.655	0.544	0.574

*Values are means ± SD*.

BMI, body mass index; EF, ejection fraction of the heart; peak HR, peak heart rate; peak VO_2_, peak oxygen consumption; peak O_2_/HR, peak fraction of oxygen to heart rate;

** *p ≤ 0.025 levels of significance effect size is displayed as η^2^*.

**Table 2:** Physiological and training-intensity related parameters during the intervention for the hypoxic group (HG) and normoxic group (NG).

**Item**	**Hypoxic group (HG)**			**Normoxic group (NG)**			**Cond. *p* (η^2^)**	**Time *p* (η^2^)**	**Int. *p* (η^2^)**
	**Tr1**	**Tr7**	**Δ Tr1-7 (%)**	**Tr1**	**Tr7**	**Δ Tr1-7 (%)**			
Watt (W)	18.53 (±6.54)	22.84 (±13.52)	23.26	23.00 (±11.51)	34.75 (±14.90)	51.09	0.058	<0.001^***^ (0.56)	0.005^***^ (0.18)
Watt/kg (W)	0.27 (±0.07)	0.32 (±0.15)	18.52	0.33 (±0.11)	0.50 (±0.13)	51.52	0.012 (0.35)	<0.001^***^ (0.53)	0.001^***^ (0.21)
Training intensity (%)	79.45 (±7.96)	80.67 (±5.80)	1.54	68.12 (±9.03)	74.43 (±8.33)	9.26	0.012^**^ (0.35)	0.059	0.739
BP systolic (mmHg)	140.47 (±22.78)	140.32 (±23.32)	−0.11	148.44 (±17.95)	141.88 (±19.94)	−4.42	0.350	0.163	0.605
BP diastolic (mmHg)	77.32 (±19.27)	76.68 (±10.24)	−0.83	82.06 (±8.21)	75.75 (±11.83)	−7.69	0.609	0.616	0.465
Velocity (km/h)	1.37 (±0.29)	1.37 (±0.36)	±0.00	1.44 (±0.29)	1.71 (±0.23)	18.75	0.070	<0.001^***^ (0.32)	0.001^***^ (0.21)
Grade (%)	0.12 (±0.46)	1.65 (±2.21)	1.275	1.13 (±1.75)	3.88 (±2.42)	243.36	0.005^**^ (0.42)	<0.001^***^ (0.46)	0.066
Walking distance (m)	263.16 (±99.12)	381.23 (±251.04)	44.87	285.63 (±105.42)	505.42 (±178.25)	76.95	0.358	<0.001^***^ (0.63)	0.492
SpO_2_ (%)	88.28 (±9.00)	90.11 (±11.47)	2.07	94.42 (±2.98)	95.15 (±2.33)	0.77	<0.001^***^ (0.72)	0.027^*^ (0.14)	0.776

*Values are means from Tr1 and Tr7 ± SD*.

Training intensity, % of peak heart rate; BP, blood pressure; SpO_2_, oxygen saturation

* p ≤ 0.05,

** p ≤ 0.025,

*** *p ≤ 0.001 levels of significance, effect size is displayed as η^2^*.

### Intervention

**Figure 1:**
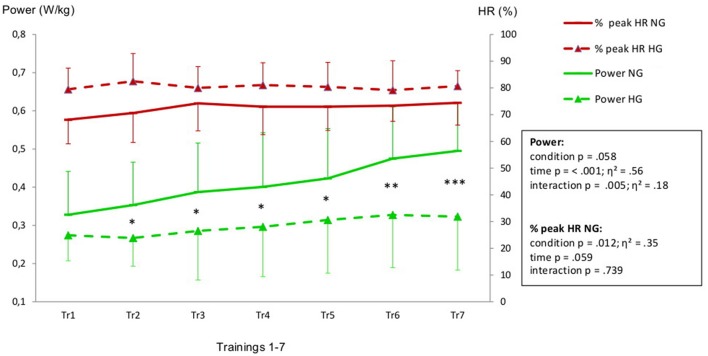
Heart rate (expressed in % of HR peak) and power values (W/kg) recorded during the training intervention (Tr1-Tr7). Legend: ^*^p ≤ 0.05, ^**^p ≤ 0.025, ^***^p ≤ 0.001 levels of significant differences between HG and NG in Tr1 to Tr7. Effect size is displayed as η^2^.

**Figure 2:**
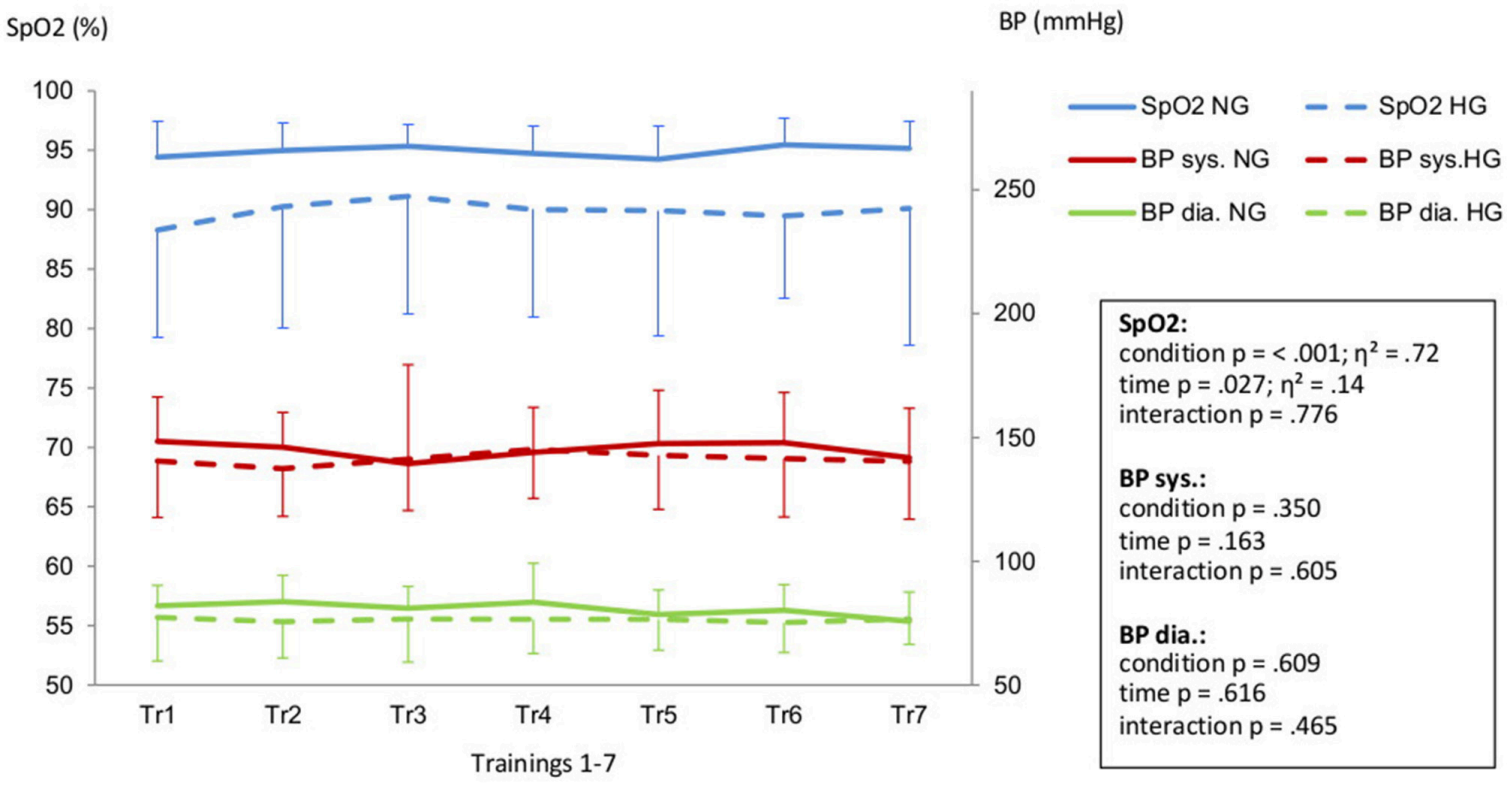
Peripheral oxygen saturation (SpO_2_) and systolic (BP sys.) aswell as diastolic blood pressure (BP dia.) in Hypoxic Group (HG) and Normoxic Group (NG) during the training intervention (Tr1-Tr7). Legend: Effect size is displayed as η^2^.

Aiming for a mean training intensity at 80% of VO_2_-peak the NG had to be pushed harder and only reached an average intensity of 74%. The HG had to be restrained to prevent them from exceeding their target HR resulting in a mean training intensity of 80%.(Figure 1). All intensity related parameters showed a significant reduction in training effort for HG as displayed in Table 2. Resting BP in both normoxic and hypoxic environments did not change over the time course of the seven training sessions (Figure 2).

## Discussion

This is the first study of endurance training in normobaric hypoxia with exclusively geriatric patients. The present study demonstrates that endurance training performed in hypoxia in geriatric patients requires less physical effort to produce an effective and comparable physiological strain (i.e., higher HR) than with similar training in normoxia. Of particular relevance in elderly, the significant reduction in mean power output by more than 25% during such hypoxic endurance training offers a gentler rehabilitation process for specific orthopedic limitations.

While endurance training could also be performed using an arm ergometer or similar ergometers depending on the patient's injury, it is noteworthy that the main goals of most geriatric patients are to get back on their feet and avoid future falls (Buchholz and Kohlmann, 2013). Combining gait training and endurance training during a stay in a rehabilitation center may improve therapy even while taking into consideration the reduced tolerance of physical training experienced by most geriatric patients. In view of this lowered tolerance for physical training, hypoxic training—which appears to be well tolerated, without provoking any complications—provides a new opportunity to enhance cardiac capacity while focusing on classical therapeutic goals (e.g., improvement in gait performance and postural stabilization) in geriatrics.

With the exception of peak power output, both groups reacted with the same non-significant physiological changes: a slightly increased VO_2_-peak, a decrease in peak O_2_/-HR and no change in EF. Evidence from related studies suggest that hypoxic exposure normalizes hypertension, improves myocardial angiogenesis or ventricular remodeling and decreases the risk for ischemic heart disease (Millet et al., 2016). Hypoxia also seems to positively affect hematological parameters as well as cognitive functioning (Schega et al., 2016). The outcomes of the present study are in line with these findings. Nevertheless, the lack of significant changes in cardiac parameters suggest that the training stimulus for our population was not challenging enough to provoke measurable cardiac adaptations to the training. However, the endurance training reflected the routines commonly employed in geriatric rehabilitation programs. Therefore, it would not have been practical to prolong the training stimulus in our patients (Meusel, 2000). Rather than improving training stimulus (e.g., volume and/or intensity), we suggest that, depending of patients' health and fitness status, implementing longer “time in hypoxia” without training may be beneficial to provoke bigger changes in cardiac and pulmonary parameters. Alternatively, more frequent interventions, with sufficient regeneration time, during a single day may prove to be more effective (Bernardi et al., 2001; Vogt et al., 2002; Foster et al., 2005). More investigations are warranted using this specific population.

An increase in maximum power output was measured in both groups after the intervention. This finding is likely due in most part to the rehabilitation routine each subject underwent during their stay. The rehabilitation intervention included physical strength training, gait training, balance training and other common physical therapy methods. The relatively lower HR observed in HG during the post-test may be an early signal of an emerging higher stroke volume and improved O_2_ utilization that has been demonstrated in other studies (Levine and Stray-Gundersen, 1997; Vissers et al., 2015; Lundby and Calbet, 2016; Moon et al., 2016). These positive outcomes may be attributed to the combined effects of the endurance training with an impactful target HR as well as the O_2_ deprivation.

There are limitations to our study. The study was conducted in geriatric patients with a variety of primary diagnoses, multiple co-morbid conditions and complex medication regimens. These circumstances naturally influenced the outcomes of the study. Nevertheless, geriatric patients typically have complex medical conditions which cannot be standardized. The complex medical histories of our patient population is a strength of this study in that the results are more likely to pertain to usual clientele served by geriatric rehabilitation programs. As stated by the World Health Organization, several diseases that our health system aims to cure in young people can be seen as a disability when it comes to old age (WHO, 2001). In order to develop an applicable hypoxic endurance training program for geriatric patients, it is important not to exclude particular medical conditions or medications. Second, the rather short hypoxic protocol was not challenging enough to provoke significant cardiopulmonary adaptations in either randomized group. Third, a larger study population may have allowed for subset analyses to identify patients with particular characteristics that would be more responsive to this intervention. Fourth, the NG did not reach the estimated 80% VO_2_ peak of training-intensity. The mean difference between NG and HG was 5.9 ± 4.2 bpm, which in our opinion does not influence the study outcome substantially. Nevertheless, the inability of the NG to reach the target training-HR validates the need for new methods to enhance training intensity.

In conclusion, the main outcome of this study demonstrated that endurance training in normobaric hypoxia demands less physical effort from the geriatric patient for the same degree of target HR response than similar training in normoxia. Our findings suggest that hypoxic endurance training is very likely to be more effective than normoxic training. Hypoxia has already been proposed to positively impact several common diseases in geriatric patients, such as hypertension, obesity, cognitive impairment and cardiopulmonary diseases (Burtscher, 2004; Millet et al., 2016; Schega et al., 2016). The great challenge for future investigations is to find a suitable and achievable training-protocol which provokes the expected beneficial adaptations for such population. Such a protocol should enhance geriatric rehabilitation while also taking into account the physical conditions that often limit the geriatric patients.

## Author contributions

SP: designing research study, acquiring data, analyzing data, conducting experiment, writing manuscript. MB, MF and HG: writing manuscript, analyzing data. LR and AE: reviewing data, writing manuscript. NN: designing research study, conducting experiment, providing material, writing manuscript.

### Conflict of interest statement

The authors declare that the research was conducted in the absence of any commercial or financial relationships that could be construed as a potential conflict of interest.

## References

[B1] Avila-Funes J. A. Pina-Escudero S. D. Aguilar-Navarro S. Gutierrez-Robledo L. M. Ruiz-Arregui L. Amieva H. (2011). Cognitive impairment and low physical activity are the components of frailty more strongly associated with disability. J. Nutr. Health. Aging 15 683–689. 10.1007/s12603-011-0111-8 21968865 PMC12878891

[B2] Bernardi L. Passino C. Serebrovskaya Z. Serebrovskaya T. Appenzeller O. (2001). Respiratory and cardiovascular adaptations to progressive hypoxia; effect of interval hypoxic training. Eur. Heart J. 22, 879–886. 10.1053/euhj.2000.2466 11350098

[B3] Borg G. A. (1982). Psychophysical bases of perceived exertion. Med. Sci. Sports Exerc. 14, 377–381. 10.1249/00005768-198205000-00012 7154893

[B4] Buchholz I. Kohlmann T. (2013). Ziele von Patienten der medizinischen Rehabilitation–Eine Übersicht zum Forschungsstand in Deutschland. Die Rehabilitation. 52, 75–85. 10.1055/s-0032-1311612 23615950

[B5] Buchner D. M. Cress M. E. de Lateur B. J. Esselman P. C. Margherita A. J. Price R. (1997). The effect of strength and endurance training on gait, balance, fall risk, and health services use in community-living older adults. J. Gerontol. Biol. Sci. Med. Sci. 52, M218–M224. 10.1093/gerona/52A.4.M218 9224433

[B6] Burtscher M. (2004). Endurance performance of the elderly mountaineer: requirements, limitations, testing, and training. Wien. Klin. Wochenschr. 116 703–714. 10.1007/s00508-004-0258-y 15628639

[B7] Cornelissen V. A. Fagard R. H. (2005). Effects of endurance training on blood pressure, blood pressure–regulating mechanisms, and cardiovascular risk factors. Hypertension 46, 667–675. 10.1161/01.HYP.0000184225.05629.51 16157788

[B8] Fiogbe E. Vassimon-Barroso V. de Medeiros Takahashi A. C. (2017). Exercise training in older adults, what effects on muscle oxygenation? A systematic review. Arch. Gerontol. Geriatr. 71, 89–98. 10.1016/j.archger.2017.03.001 28410504

[B9] Foster G. E. McKenzie D. C. Milsom W. K. Sheel A. W. (2005). Effects of two protocols of intermittent hypoxia on human ventilatory, cardiovascular and cerebral responses to hypoxia. J. Physiol. 567, 689–699. 10.1113/jphysiol.2005.091462 15975977 PMC1474187

[B10] Gatterer H. Raab C. Pramsohler S. Faulhaber M. Burtscher M. Netzer N. (2014). Effect of weekly hiking on cardiovascular risk factors in the elderly. Z. Gerontol. Geriatr. 48, 150–153. 10.1007/s00391-014-0622-0 24609428

[B11] Haufe S. (2010). Einfluss von Hypoxie und Wasserimmersion auf Stoffwechsel und Herz-Kreislaufparameter während körperlicher Aktivität. Berlin: Charité - University Medicine, dissertation.

[B12] Hayashi Y. Nagasaka S. Takahashi N. Kusaka I. Ishibashi S. Numao S. (2005). A single bout of exercise at higher intensity enhances glucose effectiveness in sedentary men. J. Clin. Endocrinol. Metab. 90, 4035–4040. 10.1210/jc.2004-2092 15840752

[B13] Jamour M. Marburger C. Runge M. Sieber C. C. Tümena T. Swoboda W. (2014). Wirksamkeit geriatrischer rehabilitation bei hochbetagten. Z. Gerontol. Geriatr. 47, 389–396. 10.1007/s00391-014-0662-5 25012107

[B14] Knuth S. (2008). Reaktionen im Bereich des kardio-vaskulären Systems auf Interventionen akuter und chronischer Hypoxie unter normobaren. Bedingungen: Deutsche Sporthochschule Köln.

[B15] Kong Z. Shi Q. Nie J. Tong T. K. Song L. Yi L. (2017). High-intensity interval training in normobaric hypoxia improves cardiorespiratory fitness in overweight chinese young women. Front. Physiol. 8:175 10.3389/fphys.2017.00175 28386234 PMC5362639

[B16] Kroidl R. F. Schwarz S. Lehnigk B. Fritsch J. (2015). Kursbuch Spiroergometrie. Pneumologie 69:164 10.1055/s-0035-1547010

[B17] Levine B. D. Stray-Gundersen J. (1997). “Living high-training low”: effect of moderate-altitude acclimatization with low-altitude training on performance. J. App. Physiol. 83, 102–112. 9216951 10.1152/jappl.1997.83.1.102

[B18] Lundby C. Calbet J. A. L. (2016). Why are high-altitude natives so strong at altitude? Maximal oxygen transport to the muscle cell in altitude natives, in Hypoxia: Translation in Progress, eds Roach R. C. Hackett P. H. Wagner P. D. (Boston, MA: Springer), 65–81. 10.1007/978-1-4899-7678-9_5 27343089

[B19] Meusel H. (2000). Grundlagen der Bewegungstherapie in der geriatrischen rehabilitation. Z. Gerontol. Geriat. 33, S035–S044. 10.1007/s003910070006 10768265

[B20] Millet G. P. Debevec T. Brocherie F. Malatesta D. Girard O. (2016). Therapeutic use of exercising in hypoxia: promises and limitations. Front. Physiol. 7:224. 10.3389/fphys.2016.00224 27375500 PMC4902009

[B21] Moon H.-W. Sunoo S. Park H.-Y. Lee D.-J. Nam S.-S. (2016). Effects of various acute hypoxic conditions on metabolic parameters and cardiac function during exercise and recovery. Springer Plus 5:1294. 10.1186/s40064-016-2952-4 27547668 PMC4977266

[B22] Netzer N. C. Chytra R. Kupper T. (2008). Low intense physical exercise in normobaric hypoxia leads to more weight loss in obese people than low intense physical exercise in normobaric sham hypoxia. Sleep Breath. 12, 129–134. 10.1007/s11325-007-0149-3 18057976 PMC2276561

[B23] Oster P. Pfisterer M. Schuler M. Hauer K. (2005). Körperliches training im Alter. Z. Gerontol. Geriatr. 38, i10–i13. 10.1007/s00391-005-1103-2 16189729

[B24] Pandey A. Kitzman D. W. Brubaker P. Haykowsky M. J. Morgan T. Becton J. T. (2017). Response to endurance exercise training in older adults with heart failure with preserved or reduced ejection fraction. J. Am. Geriatr. Soc. 10.1111/jgs.14867. [Epub ahead of print]. PMC5555804 28338229

[B25] Piehl Aulin K. Svedenhag J. Wide L. Berglund B. Saltin B. (1998). Short-term intermittent normobaric hypoxia–haematological, physiological and mental effects. Scand J. Med. Sci. Sports. 8, 132–137. 10.1111/j.1600-0838.1998.tb00182.x 9659672

[B26] Pokan R. Benzer W. Gabriel H. Hofmann P. Kunschitz E. Mayr K. (2009). Kompendium der kardiologischen Prävention und Rehabilitation. Vienna: Springer Vienna.

[B27] Poterba J. (2016). Economic implications of demographic change. Bus. Econ. 51, 3–7. 10.1057/be.2016.5

[B28] Schega L. Peter B. Brigadski T. Leßmann V. Isermann B. Hamacher D.. (2016). Effect of intermittent normobaric hypoxia on aerobic capacity and cognitive function in older people. J. Sci. Med. Sport 19, 941–945. 10.1016/j.jsams.2016.02.012 27134133

[B29] Schipfer S. Neubaer S. Mühlfeldner A. Huber R. Fischer R. (2008). Trainingseffekte unter normobarer Hypoxie auf Patienten mit metabolischem Syndrom. Pneumologie 62:397 10.1055/s-2008-107413218623626

[B30] Swain D. P. Franklin B. A. (2006). Comparison of cardioprotective benefits of vigorous versus moderate intensity aerobic exercise. Am. J. Cardiol. 97, 141–147. 10.1016/j.amjcard.2005.07.130 16377300

[B31] Vaitkevicius P. V. Ebersold C. Shah M. S. Gill N. S. Katz R. L. Narrett M. J.. (2002). Effects of aerobic exercise training in community-based subjects aged 80 and older: a pilot study. J. Am. Geriatr. Soc. 50, 2009–2013. 10.1046/j.1532-5415.2002.50613.x 12473013

[B32] Ventura-Clapier R. Mettauer B. Bigard X. (2007). Beneficial effects of endurance training on cardiac and skeletal muscle energy metabolism in heart failure. Cardiovasc. Res. 73, 10–18. 10.1016/j.cardiores.2006.09.003 17045979

[B33] Vissers H. Stratton W. G. Hamann J. Erepezko J. H. Voyles P. M. Mao X. (2015). Altitude Training and Its Effects on the Human Body. River Falls, WI: University of Wisconsin.

[B34] Vogt M. Flück M. Hoppeler H. (2002). ‘Living low–Training high’: Eine effektive Höhentrainingsmethode zur Verbesserung der sportlichen Leistungsfähigkeit von trainierten Athleten, in Jahrbuch 2002 der Österreichischen Gesellschaft für Alpin- und Höhenmedizin, ed Sumann G. (Innsbruck: Raggl), 89–104.

[B35] WHO (2001). International Classification of Functioning, Disability and Health: ICF. Geneva: World Health Organization.

